# Fenretinide in cancer therapy and chemoprevention: past, present and future

**DOI:** 10.20517/cdr.2025.210

**Published:** 2026-06-05

**Authors:** Paola Verachi, Federica Francescangeli, Rosanna Dattilo, Alessandro Vici, Rachele Rossi, Elena Landolfo, Maria Laura De Angelis, Ann Zeuner

**Affiliations:** Department of Oncology and Molecular Medicine, Istituto Superiore di Sanità, Rome 00161, Italy.

**Keywords:** Fenretinide, 4-HPR, tumor dormancy, nanoformulations, chemoprevention, cancer stem cells

## Abstract

Fenretinide [N-(4-hydroxyphenyl) retinamide, 4-HPR] is a synthetic retinoid known for decades for its antitumor activity and favorable toxicological profile. *In vitro* and *in vivo* studies have demonstrated that fenretinide has broad-spectrum anticancer potential, eliciting multiple biological effects and acting as a multi-target compound that influences both tumor cells and the tumor microenvironment. However, the clinical application of fenretinide has been hampered by unfavorable physicochemical properties, such as poor solubility and low bioavailability, leading to highly variable outcomes in clinical studies. The development of novel fenretinide nanoformulations based on drug encapsulation in nanomicelles has helped overcome bioavailability limitations and improve anticancer efficacy in preclinical studies, thereby paving the way for new clinical opportunities. Moreover, recent studies have shown that fenretinide restrains the cancer stem cell compartment and promotes tumor cell dormancy, suggesting its potential use in dormancy-inducing strategies aimed at preventing and/or controlling metastatic disease. Here, we provide an overview of fenretinide’s mechanisms of action in cancer cells, summarize the available clinical evidence and discuss advances achieved with novel formulations. Finally, we discuss the potential integration of fenretinide at different stages of clinical management, with particular emphasis on its prospective use as a chemopreventive strategy or as a therapeutic option for disease control.

## INTRODUCTION

Retinoids are a class of natural and synthetic compounds structurally related to vitamin A. They have long been studied for their effects on crucial biological processes, such as cell growth, differentiation and metabolism. In addition, retinoids have a well-established reputation for their therapeutic potential across various tumor types, primarily through the modulation of nuclear retinoic acid receptors (RARs) and retinoid X receptors (RXRs), resulting in growth inhibition and induction of cancer cell apoptosis. Moreover, retinoids have attracted attention for their ability to promote cellular differentiation in multiple tumor types^[1,2]^. Notably, the concept of differentiation therapy originated from the observation that, in acute promyelocytic leukemia (APL), leukemic blasts can be induced to differentiate into mature granulocytes following treatment with all-trans retinoic acid (ATRA)^[3-5]^. However, despite the success of APL treatment protocols, the broader clinical application of retinoids remains limited by their high toxicity and poor bioavailability.

**Figure 1:**
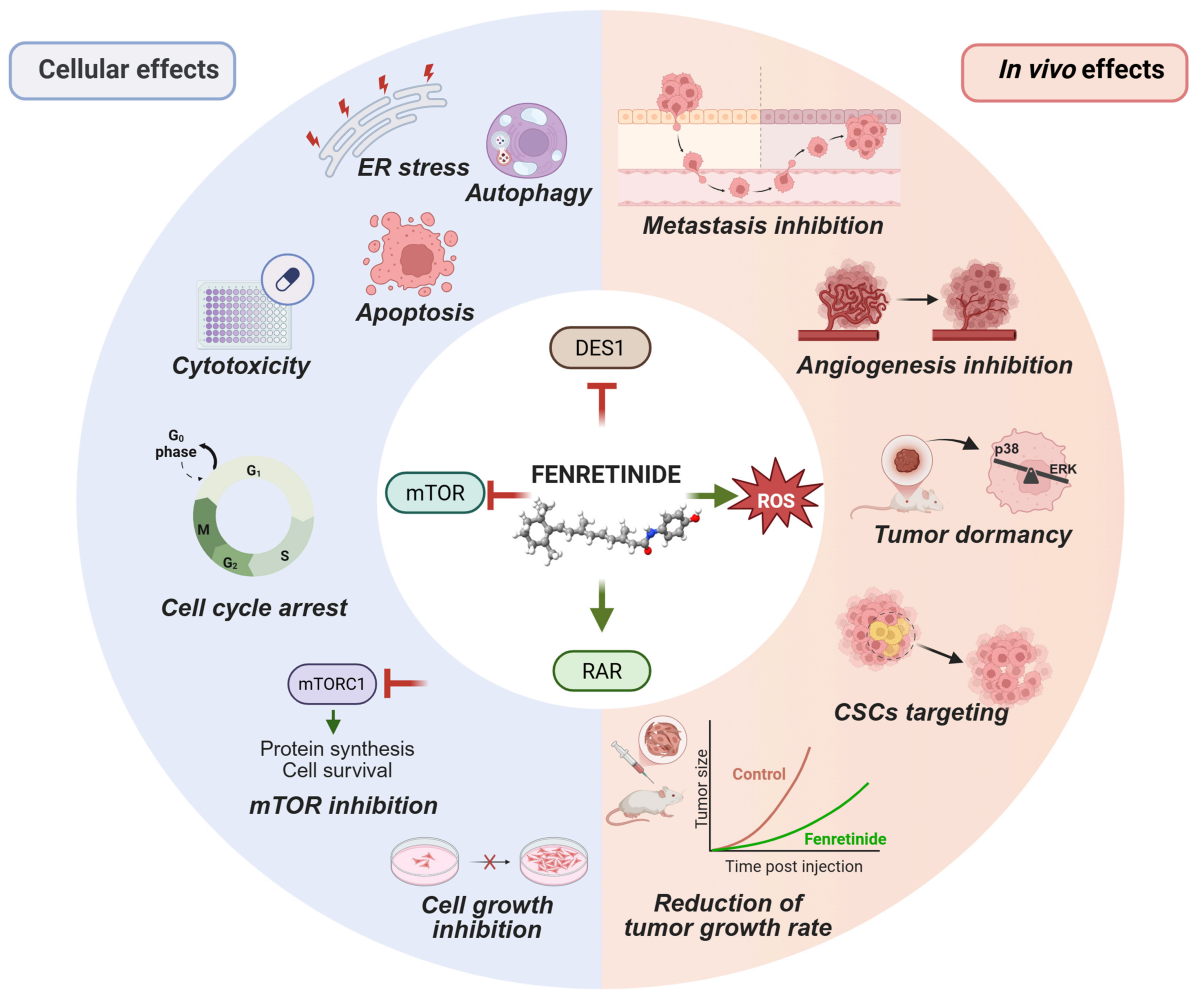
Multifaceted antitumor mechanisms of fenretinide. Cellular (left) and *in vivo* (right) antitumor effects of fenretinide are driven by multiple mechanisms (center), such as direct interaction with RAR, suppression of the mTOR pathway, generation of ROS and generation of lipid second messengers (the latter by DES1 inhibition). Red T-bars indicate inhibition. Green arrows indicate activation. Created in BioRender. Verachi, P. (2026) https://BioRender.com/5r4zhea. RAR: Retinoic acid receptor; mTOR: mechanistic target of rapamycin; ROS: reactive oxygen species; DES1: dihydroceramide desaturase 1; ER: endoplasmic reticulum; mTORC1: mTOR complex 1; ERK: extracellular signal-regulated kinase; CSCs: cancer stem cells.

Among synthetic retinoids, fenretinide [N-(4-hydroxyphenyl) retinamide, 4-HPR] has emerged as a particularly promising antitumor agent due to its unique pharmacological profile. Originally produced in the USA in 1960, this compound is a synthetic amide derived from ATRA but behaves as an atypical retinoid with both RAR-dependent and RAR-independent activities^[6]^. Early studies demonstrated that, unlike ATRA, which primarily promotes cellular differentiation, fenretinide acts by inhibiting cell growth and inducing apoptosis, even in ATRA-resistant cell lines^[6]^. Indeed, it has been demonstrated that the pro-apoptotic activity of fenretinide involves multiple RAR-independent mechanisms, including the generation of mitochondrial reactive oxygen species (ROS) and the activation of caspases [Figure 1]^[7,8]^. Moreover, by targeting dihydroceramide desaturase 1 (DES1), fenretinide induces the accumulation of dihydroceramide (dhCer), a bioactive lipid with pleiotropic effects on several cellular functions [Figure 1]^[8-10]^. Beyond its pro-apoptotic activity, fenretinide exerts antitumor effects by modulating key biological processes involved in cell proliferation, biosynthesis and survival, such as suppression of the mechanistic target of rapamycin (mTOR) pathway, induction of dormancy and autophagy^[8,11-13]^. In addition, fenretinide has attracted considerable attention for its ability to affect cancer stem cells (CSCs) [Figure 1], which play a pivotal role in tumor progression, relapse, metastasis and resistance to radio- and chemotherapy^[14-18]^. Interestingly, fenretinide has been reported to exhibit anti-angiogenic properties and to modulate the tumor microenvironment (TME), leading to impaired tumor growth^[19-22]^. The main anticancer effects of fenretinide are summarized in Figure 1 and Table 1. Cancer constitutes one of the leading causes of morbidity and mortality worldwide^[23]^. Conventional therapies such as chemotherapy and radiotherapy often lead to a temporary response, ultimately promoting therapy resistance and subsequent disease progression^[24-27]^. One of the major causes of therapy failure is intra-tumor heterogeneity^[28-30]^, which results from the interplay between cancer cells and the TME. Cancer cells undergo continuous genetic, epigenetic and metabolic alterations that further increase tumor heterogeneity^[8,24]^. Moreover, intra-tumor heterogeneity evolves over time and is exacerbated in response to therapy^[30,31]^. This dynamic and heterogeneous nature of cancer limits the efficacy of therapies targeting a single molecular pathway, which often fail to achieve complete tumor eradication due to the high plasticity and clonal adaptability of cancer cells. Multi-target treatment strategies proposed in recent years offer several advantages, such as the ability to overcome clonal heterogeneity, to decrease the emergence of multidrug resistance and to reduce systemic toxicity^[32,33]^. In this context, fenretinide has emerged as a promising therapeutic candidate due to its broad-spectrum activity and its ability to simultaneously modulate multiple pathways involved in tumor progression, along with its favorable toxicological profile. Indeed, fenretinide exhibits selective cytotoxicity toward malignant cells while sparing normal tissues, thus minimizing the toxic effects typically associated with retinoids^[6,12,14,18,34-37]^. Moreover, fenretinide does not accumulate in the liver, implying a reduction of hepatic toxicity, as consistently demonstrated in preclinical studies^[6,12,14]^. In addition, fenretinide has shown well-tolerated safety profiles in early-phase clinical trials, further supporting its potential integration into combination treatment regimens^[38-40]^. Despite decades of evidence supporting its antitumor efficacy and favorable toxicity profile, the clinical use of fenretinide has been limited by its challenging physicochemical properties^[41,42]^. Unlike other retinoids such as ATRA, which achieve adequate systemic exposure despite rapid clearance, fenretinide is characterized by long elimination half-life and tissue-specific accumulation, but exhibits markedly poor bioavailability due to its lipophilicity and low aqueous solubility^[43]^. These pharmacokinetic limitations required the use of high-dose regimens to achieve therapeutic plasma concentrations in earlier clinical trials, thereby compromising drug tolerability and reducing patient compliance^[44]^. In recent years, several laboratories have sought to improve fenretinide bioavailability by developing novel formulations with improved solubility and enhanced antitumor efficacy. This review outlines the mechanisms underlying the antitumor effects of fenretinide and discusses preclinical evidence supporting its potential use in cancer treatment and chemoprevention. Finally, we summarize the clinical findings across different cancer types, highlighting advances achieved with the use of novel fenretinide formulations developed in recent years.

**Table 1:** Summary of antitumor effects of fenretinide

**Mechanism**	**Description**	**Key pathways/molecules**
Apoptosis induction	Induces apoptosis through RARβ/γ	RARβ/γ, Nur77, Bcl-2
	Induces apoptosis through RAR-independent pathways involving ROS generation, Bcl-2 conformational change, and caspase activation	Bcl-2, ↑ROS, caspase-3
ROS generation and mitochondrial stress	Promotes ROS generation and CytC release; disrupts PKCδ-CytC signalosome	↑ROS, CytC, PKCδ
Bioactive lipids modulation (Cer/dhCer)	Inhibits DES1, increases dhCer levels, triggers ER stress, autophagy, and cell cycle delay, contributing to cytotoxicity	↓DES1, ↑dhCer, ER stress, mTORC1
mTOR pathway inhibition	Directly binds to mTOR ATP pocket and inhibits mTORC1/2, reducing cell proliferation and survival signaling	↓mTORC1, ↓mTORC2, PI3K/AKT
Autophagy induction	Activates autophagy in a context- and dose-dependent manner; can promote survival or cell death depending on ROS levels and apoptotic efficiency	↑dhCer, ↑ROS, ↑UPR, ↑LC3/LC3-II, p38, PI3K/AKT/mTORC
Targeting CSCs	Targets CSCs by inducing ROS and ER stress, inhibiting NF-κB and Wnt/β-catenin signaling, and promoting cell cycle arrest and dormancy	↑ROS, ↓NF-κB, ↓Wnt/β-catenin, ↑p16, ↑p-p38
Cell cycle arrest/dormancy	Induces G0/G1, G1/S, or G2/M arrest depending on cell type; modulates cyclins, CDKs, p16, p-p38, and pERK to induce cellular dormancy	↑p16, ↑p-p38, ↓pERK, ↓CDK1, ↓CDK4, ↓cyclin D1, ↓RB
TME modulation	Suppresses angiogenesis, inhibits M2 macrophage polarization and cell-ECM interactions	↓VEGF/VEGFR, ↓JNK/MMP-2, ↑BMP-2/MIC-1, STAT6

RAR: Retinoic acid receptor; ROS: reactive oxygen species; Bcl-2: B-cell lymphoma 2; Nur77: nuclear receptor 4A1; CytC: cytochrome c; PKCδ: protein kinase C delta; Cer: ceramide; dhCer: dihydroceramide; DES1: dihydroceramide desaturase 1; ER: endoplasmic reticulum; mTOR: mechanistic target of rapamycin; mTORC1: mTOR complex 1; mTORC2: mTOR complex 2; PI3K: phosphoinositide 3-kinase; AKT: protein kinase B; UPR: unfolded protein response; LC3: microtubule-associated protein 1A/1B-light chain 3; LC3-II: lipid modified form of LC3; CSCs: cancer stem cells; NF-κB: nuclear factor kappa-light-chain-enhancer of activated B cells; p-p38: phosphorylated p38; CDKs: cyclin-dependent kinases; pERK: phosphorylated extracellular signal-regulated kinase; RB: retinoblastoma; TME: tumor microenvironment; VEGF: vascular endothelial growth factor; VEGFR: vascular endothelial growth factor receptor; JNK: c-Jun N-terminal kinase; MMP-2: matrix metalloproteinase-2; BMP-2: bone morphogenetic protein 2; MIC-1: macrophage inhibitory cytokine-1; STAT6: signal transducer and activator of transcription 6.

## BIOLOGICAL ACTIVITIES OF FENRETINIDE IN CANCER

### RAR-dependent and independent apoptotic effects

Typical retinoids exert their antitumor activity mainly through the activation of RARs and RXRs, resulting in decreased expression of anti-apoptotic B-cell lymphoma 2 (Bcl-2) family genes^[45]^. In contrast, fenretinide’s pro-apoptotic effect involves diverse signaling mediators activated by RAR-independent mechanisms, such as ROS, lipid second messengers, and stress-related proteins^[7-10,46]^. Additional mechanisms contributing to fenretinide’s antitumor activity include induction of autophagy, cell cycle arrest and/or slowing, reduction of the CSCs content, depression of biosynthesis processes and inhibition of survival pathways^[8,12-18,47]^. Transactivation assays have demonstrated that fenretinide acts as a potent transactivator of RARγ and a moderate activator of RARβ, while exhibiting no interaction with RARα and RXRα. This selective activation profile may explain its specific biological activities and favorable pharmaceutical properties^[48]^. A study on hepatocellular carcinoma (HCC) cell lines demonstrated that the pro-apoptotic effect of fenretinide is mediated by direct activation of RARβ, whose expression also determines the susceptibility of HCC cells to fenretinide-induced apoptosis^[49]^. In a subsequent study, the same authors identified nuclear receptor 4A1 (Nur77) as an additional mediator of fenretinide-induced apoptosis. Specifically, in Huh7 cells fenretinide promoted the translocation of Nur77 from the nucleus to the mitochondria, allowing its binding to Bcl-2 and inducing a conformational change of this protein toward a pro-apoptotic state^[50]^. Accordingly, Nur77 knockdown prevented fenretinide-induced DNA double-strand breaks and caspase-3 cleavage^[50]^. Similarly, in acute myeloid leukemia (AML) cells, fenretinide induced the translocation of Nur77 from the nucleus to the mitochondria, where it bound Bcl-2^[51]^. Another study in HCC cells showed that Nur77 directly interacted with RARβ and that the induction of Nur77 expression was dependent upon the presence of RARβ, with which it co-localized in the cytoplasm^[52]^. Consistent with these findings, in human neuroblastoma cells fenretinide-induced apoptosis was blocked by two RARβ/γ-specific antagonists, but not by a RARα-specific antagonist^[53]^. However, a study on human epidermoid carcinoma A431 cells showed that treatment with RAR antagonists and caspase inhibitors provided only partial protection against fenretinide, suggesting that fenretinide-mediated apoptosis is a complex process that involves both RAR-dependent and RAR–independent pathways^[54]^. Abnormal ROS generation plays a pivotal role in apoptosis signaling, leading to an increase in mitochondrial membrane permeability and subsequent release of pro-apoptotic proteins such as cytochrome c (CytC), which promotes cell death via caspase activation^[55]^. Moreover, aberrant ROS production can lead to the generation of bioactive lipids such as ceramides, which trigger mitochondrial-mediated apoptosis through direct disruption of mitochondrial integrity^[56,57]^. Elevated levels of ROS, particularly hydroperoxides, have been observed in a variety of cancer cell types following exposure to fenretinide. The ability of antioxidants to counteract fenretinide-induced apoptosis further supports a crucial role for oxidative stress in its cytotoxic effects^[7]^. In multiple cancer cell lines, fenretinide has been shown to induce CytC release and to impair mitochondrial oxidative phosphorylation (OXPHOS), indicating that fenretinide-induced ROS generation mainly takes place in the mitochondria^[7,58]^. Consistent with this mechanism, fenretinide has been reported to act as a functional antagonist of retinol in mitochondrial signaling by disrupting the protein kinase Cδ (PKCδ)-CytC signalosome, a crucial OXPHOS regulator^[55]^.

### Role of lipid second messengers in fenretinide-induced cytotoxicity

Sphingolipids are membrane lipids that, besides their structural roles, function as key signaling molecules regulating cell survival, metabolism, and stress responses^[59]^. Ceramide (Cer), a bioactive lipid primarily produced through *de novo* sphingolipid biosynthesis, has attracted considerable attention for its ability to promote apoptosis in response to cellular stress^[60]^. In various cancer cell models, fenretinide has been shown to increase Cer levels independently of caspase activation^[61,62]^, suggesting that it regulates *de novo* Cer biosynthesis and/or sphingomyelin hydrolysis. Several studies have demonstrated that fenretinide inhibits DES1, the enzyme that catalyzes the conversion of dhCers into Cer^[8,9,63-66]^. In human neuroblastoma SMS-KCNR cells, DES1 silencing resulted in a marked accumulation of endogenous dhCers and subsequent inhibition of cell growth with cell cycle arrest in G0/G1. Moreover, fenretinide treatment inhibited desaturase activity in a dose-dependent manner without affecting messenger RNA (mRNA) or DES1 protein levels, suggesting that fenretinide may act as a direct enzymatic inhibitor^[63]^. Consistent with these findings, treatment of lung and colorectal cancer (CRC) spheroid cultures with fenretinide led to significant accumulation of d18-dhCer, while pre-treatment with myriocin, an inhibitor of *de novo* sphingolipid biosynthesis, partially protected cells from fenretinide-induced cell death^[8]^. Furthermore, a lipidomic study has shown that fenretinide-induced dhCers accumulation promotes autophagy by inducing autophagosome formation in prostate cancer cells^[64]^. Subsequent studies demonstrated a role of dhCers in autophagy by showing that elevated dhCers levels lead to delayed G1/S cell cycle progression and endoplasmic reticulum (ER) stress induction^[60]^. In line with this, a study on glioma cells revealed that DES1 inhibition increases the dhCer/Cer ratio in the ER membrane, thereby triggering ER stress and inhibiting the protein kinase B (Akt)/mTOR complex 1 (mTORC1) pathway, ultimately leading to autophagy^[67]^.

### Fenretinide-mediated mTOR suppression and downstream biological effects

The serine/threonine kinase mTOR is crucial for the regulation of cell survival, metabolism, cell growth, and protein biosynthesis, and is aberrantly upregulated in more than 70% of cancers, contributing to tumorigenesis^[68]^. Previous studies have shown that fenretinide targets the phosphoinositide 3-kinase (PI3K)/Akt/mTOR signaling pathway and that Akt phosphorylation at Ser473 is implicated in fenretinide-induced apoptosis^[69,70]^. In 2012 mTOR was identified for the first time as a direct molecular target of fenretinide in both *in vitro* and *in vivo* models^[11]^. Specifically, molecular docking analyses showed that fenretinide binds to the ATP-binding pocket of mTOR, forming hydrogen bonds with Ser2165 and Lys2187, as well as hydrophobic interactions with key residues for the binding of ATP. Direct inhibition of mTOR suppressed the activity of both mTORC1 and mTOR complex 2 (mTORC2), resulting in reduced proliferation of lung cancer cells *in vitro* and decreased tumor growth in mice^[11]^. Interestingly, mTOR knockout in A549 cells decreased their sensitivity to fenretinide, suggesting that mTOR signaling is essential for their antitumor activity. However, no obvious apoptosis was observed in JB6 C141 cells and A549 cells after exposure to fenretinide at concentrations required to inhibit mTOR and cell proliferation, suggesting that mTOR targeting by fenretinide primarily contributes to its antiproliferative activity rather than to induction of apoptosis^[11]^. Consistent with those findings, a recent study on human epidermal growth factor receptor 2 (HER2)/neu (neuT) transgenic mice, a model of spontaneously metastatic breast cancer (BC), demonstrated that fenretinide treatment decreased mTOR pathway activation and cell cycle progression, inhibiting the growth of primary and metastatic tumors in the absence of overt apoptosis^[12]^.

### Autophagic signals induced by fenretinide: a double-edged sword?

Autophagy is a tightly regulated catabolic process characterized by the degradation of cellular components, reorganization of intracellular membranes and vesicles, and lysosomal activity^[71]^. In recent years, autophagic cell death has been increasingly recognized as a key component of cellular stress responses and tumorigenesis. Indeed, cancer cells can activate autophagy as a survival mechanism in response to stressors such as radiation and chemotherapy, thus contributing to chemoresistance^[13]^. However, autophagy can also promote cell death, as excessive autophagic activity can lead to the digestion of the entire cell^[72]^. As mentioned in section “Role of lipid second messengers in fenretinide-induced cytotoxicity”, fenretinide inhibits DES1, leading to increased dhCer levels. Mechanistically, alterations in dhCer levels modify the lipid composition of intracellular membranes, triggering stress responses, such as oxidative and ER stress, that accompany or precede autophagy signals^[60]^. However, whether fenretinide-induced autophagy is a pro-survival or a pro-death response remains a matter of debate. Fenretinide’s ability to induce autophagy is context-dependent, with some studies supporting pro-survival effects and others linking autophagic signals to cytotoxic effects. For example, a study showed that fenretinide-induced ROS production led to the unfolded protein response (UPR) in cancer cells, and the efficiency of this response determined cell fate. An impaired UPR leads to insufficient autophagosome formation and promotes apoptosis, whereas a complete UPR response enhances autophagic clearance of protein aggregates, favoring cell survival^[73]^. Fenretinide has been reported to trigger autophagic cell death when apoptotic pathways are deregulated, as in the case of caspase-3-defective Michigan Cancer Foundation-7 (MCF-7) cells. Conversely, an isogenic cancer cell line with functional caspase-3 did not exhibit autophagy features following fenretinide treatment^[13]^. In CRC and lung cancer spheroids, fenretinide treatment increased the expression of both microtubule-associated protein 1A/1B-light chain 3 (LC3) and its lipid-modified form LC3-II, and the autophagy inhibitor 3-methyladenine attenuated its cytotoxic effects, indicating that autophagic signals contribute to fenretinide-induced cell death in these models^[8]^. A recent review has proposed that the cell fate balance between apoptosis and autophagy following fenretinide exposure is dose-dependent and strictly linked to ROS levels. High concentrations of fenretinide induce elevated ROS levels, activating the p38-dependent apoptotic pathway and suppressing the PI3K/AKT/mTORC, DES1 and RARβ-Nur77 pathways, whereas lower concentrations lead to a modest increase of ROS levels, activating autophagy pathways^[41]^.

### Fenretinide and CSCs

**Figure 2:**
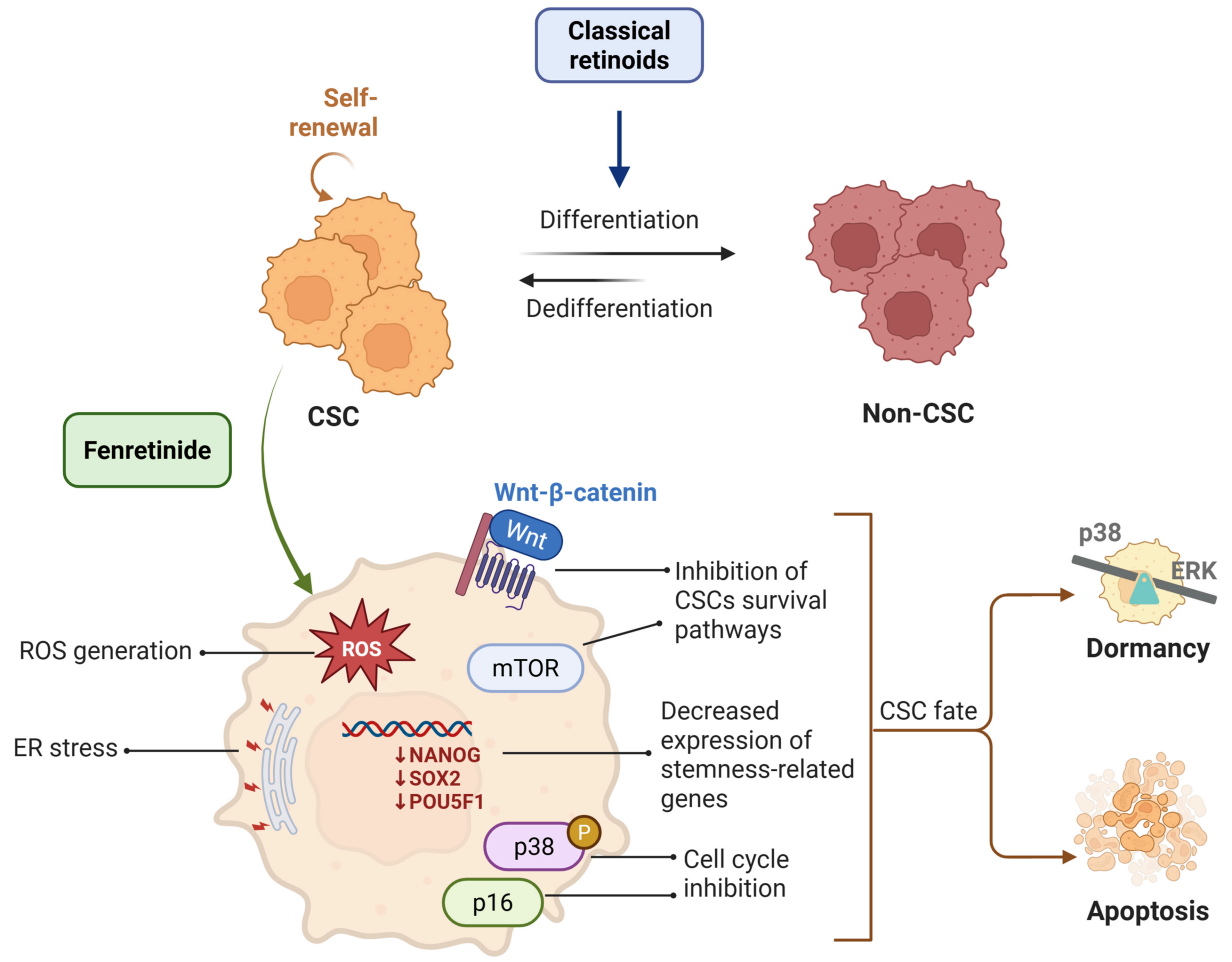
Mechanisms of CSC targeting by fenretinide. While classical retinoids are known to promote the differentiation of CSCs, fenretinide can reduce the content of this population by multiple mechanisms, i.e., by promoting apoptosis or inducing dormancy. Created in BioRender. Verachi, P. (2026) https://BioRender.com/qawlsjb. CSC: Cancer stem cell; ROS: reactive oxygen species; ER: endoplasmic reticulum; mTOR: mechanistic target of rapamycin; ERK: extracellular signal-regulated kinase.

CSCs were first identified in pioneering studies on AML, which demonstrated that only a rare subset of leukemic cells expressing stemness-associated markers was capable of initiating disease in immunodeficient mice^[74,75]^. Subsequent studies identified CSCs in a variety of solid tumors, including brain^[76]^, prostate^[77]^, colorectal^[78]^, pancreatic^[79]^, breast^[80]^ and lung cancer^[81]^. CSCs are characterized by self-renewal capacity, tumor-initiating potential and intrinsic resistance to cytotoxic agents, and therefore constitute key drivers of tumor relapse and therapeutic failure^[24]^. They can evade the toxic effects of chemotherapy through a variety of mechanisms, including activation of pro-survival pathways such as PI3K/Akt/mTOR, upregulation of anti-apoptotic factors, and entry into a chemoresistant state of dormancy^[24,82,83]^. Moreover, it has been demonstrated that CSCs can exploit autophagy as a response to stress, thereby promoting chemoresistance and subsequent disease progression^[84,85]^. As mentioned in the introduction, the effectiveness of differentiating therapy was first demonstrated with ATRA in the targeted treatment of APL^[4,86,87]^, paving the way for the development of anti-CSCs therapeutic strategies. However, it has been demonstrated that differentiated cells can revert to an undifferentiated phenotype over time, highlighting the limitations of this approach^[88-91]^. A recent study proposed the use of fenretinide as a potential alternative therapeutic strategy for APL in cases of resistance after protracted treatment with ATRA. In particular, the authors of this study demonstrated that the antitumor activity of fenretinide relies on apoptosis induction rather than differentiation^[92]^. Consistent with this mechanism, a previous study in AML showed a selective cytotoxic activity on CD34^+^ leukemic stem/progenitor cells (LSCs) without affecting the normal counterparts^[18]^. LSCs predominantly exhibit a quiescent phenotype that renders them resistant to conventional chemotherapeutic agents^[18]^. Notably, fenretinide was found to inhibit the clonogenic capacity of primary AML CD34^+^ cells *in vitro* and reduce the engraftment of primary and secondary recipients *in vivo*, suggesting a specific effect on tumor-initiating cells. Mechanistically, fenretinide induces ROS production and inhibits the nuclear factor kappa-light-chain-enhancer of activated B cells (NF-κB) and Wnt/β-catenin pathways, both of which support LSCs survival and chemoresistance^[18]^ [Figure 2]. Similar findings have been reported in chronic myeloid leukemia, where fenretinide preferentially targets LSCs through the oxidative/ER stress-mediated pathway^[15]^. Additional studies have demonstrated that fenretinide is also effective against CSCs in various solid tumors, such as medulloblastoma, colon, breast, and ovarian cancer^[8,16,17,47]^. Using tumor spheroids enriched in stem-like cells, fenretinide was shown to preferentially target CSCs through ROS induction, ER stress, and inhibition of cell-cycle progression^[16,17]^. Notably, low levels of ROS in CSCs are associated with enhanced tumorigenicity and resistance to radiotherapy^[93]^, suggesting that the stress-inducing effects of fenretinide may underlie its activity against these cells. In addition, fenretinide displayed marked efficacy against patient-derived CSCs both *in vitro* and in CSCs-derived xenografts of lung cancer, melanoma and CRC^[8,14]^. Its anti-tumor activity correlated with reduced cell proliferation, induction of apoptosis, modulation of lipid metabolism, and downregulation of CSCs-specific markers. In primary lung cancer spheroids, fenretinide decreased the expression of stemness-related genes such as *NANOG, SOX2* and *POU5F1*, whereas in CRC spheroids it reduced Wnt signaling activity^[8]^ [Figure 2]. In addition, reverse-phase proteomic analysis of fenretinide-treated primary lung cancer spheroids revealed a widespread repression of the mTOR pathway^[8]^ [Figure 2]. Consistently, a recent study in neuT mice showed that treatment with fenretinide prevented both initiation and progression of metastases through a combined induction of antiproliferative signals and inhibition of the mTOR pathway^[12]^. Taken together, these findings strongly suggest that fenretinide preferentially targets CSCs through multiple mechanisms, including induction of ROS production, ER stress, and inhibition of key pathways that sustain CSCs survival and chemoresistance [Figure 2].

### Cell cycle inhibition and dormancy-related effects of fenretinide

Several studies have demonstrated that fenretinide can induce cell cycle arrest across various cancer types. However, the reported effects on the cell cycle are not consistent and appear to depend on both the experimental context and the tumor type. In a study on human CRC spheroids, treatment with fenretinide increased the proportion of cells in the G1 and G2 phases, with a corresponding decrease of the proportion of cells in the S phase. Moreover, fenretinide-treated cells exhibited downregulation of genes involved in G1/S and G2/M cell cycle transitions^[16]^. In medulloblastoma cells, the impact of fenretinide on the cell cycle varied depending on the dose and the cell line tested. Specifically, fenretinide induced a dose-dependent increase in the proportion of DAOY cells in the S-phase, whereas ONS-76 cells showed an increased rate of apoptosis. Consistently, treatment with fenretinide caused a downregulation of cyclin D1 and cyclin-dependent kinase 4 (CDK4) in DAOY cells, while in ONS-76 only the highest dose tested resulted in a decrease in CDK4 expression. Notably, in both cell lines fenretinide induced checkpoint kinase 2 (CHK2) phosphorylation, which was associated with cell cycle arrest in S-phase^[47]^. Similarly, in AML HL-60 cells fenretinide downregulated cyclin-dependent kinase 1 (CDK1) and retinoblastoma (RB) expression, while increasing phosphorylated RB (p-RB), ultimately leading to cell cycle arrest in the S-phase^[51]^. In A2780 ovarian carcinoma cells, treatment with fenretinide caused a modest arrest in the G1 phase without altering the expression of the G1 regulators cyclin B1, cyclin D1, and cyclin E. Interestingly, the same study demonstrated that 4-oxo-fenretinide, an active metabolite of fenretinide, was able to induce G2/M arrest and alter the expression of the cell cycle regulatory proteins CDC25C and phosphorylated CDK1 (p-CDK1)^[94]^. In primary lung and CRC spheroids, a novel fenretinide nanoformulation (NanoFEN) decreased the expression of cell cycle-related factors while increasing the levels of p16 and phosphorylated p38 (p-p38), suggesting a block at the G0/early G1 phase of the cell cycle [Figure 2]^[8]^. This effect was further supported by the use of the mVenus p27K^-^ reporter, which labels cells in the G0 phase and at the G0/G1 transition^[95]^. NanoFEN treatment markedly increased the number of Venus^+^ cells, indicating that it promotes entry into, or retention of, a quiescent state^[8]^. These findings were subsequently validated *in vivo* in neuT mice treated with an orally bioavailable fenretinide nanoformulation (Bio-nFeR)^[12]^. Bio-nFeR significantly reduced the growth of primary tumors and decreased the expression of the proliferation marker Ki67. Similarly, it reduced the number and size of lung metastases, which exhibited decreased levels of Ki67 and proliferating cell nuclear antigen (PCNA). In addition, primary tumors treated with Bio-nFeR showed a strongly increased expression of p-p38 and a decreased amount of phosphorylated extracellular signal-regulated kinase (pERK), where the low pERK/p-p38 ratio represents a key feature of cancer dormancy [Figure 2]^[96]^. Treatment with Bio-nFeR also resulted in suppression of the mTOR pathway in murine breast tumors, consistent with the notion that mTOR inhibition promotes a state of cellular “hibernation” in both stem cells and cancer cells^[82]^. Taken together, these results indicate that in neuT mice, fenretinide counteracts tumor growth and metastatic progression by inducing cellular dormancy alongside suppression of metabolic and biosynthetic pathways^[12]^. In recent years, the concept of therapeutically inducing tumor dormancy has gained increasing attention as a strategy to transform cancer into a clinically manageable, non-aggressive disease^[97]^. Emerging preclinical and clinical evidence supports the feasibility of dormancy-based therapeutic approaches, particularly in the context of disseminated tumor cells (DTCs) and minimal residual disease^[97-99]^. In this framework, the ability of novel fenretinide formulations to induce tumor dormancy highlights their potential as chemopreventive agents in BC and possibly in other tumors. When considering fenretinide-induced dormancy, it is important to distinguish between cellular dormancy and tumor mass dormancy. Cellular dormancy refers to individual cancer cells entering a reversible G0/G1 arrest while maintaining metabolic activity without proliferation^[1,7,8]^, whereas tumor mass dormancy is defined as a dynamic equilibrium between cellular proliferation and apoptosis that results in stable tumor size without net expansion^[100,101]^. In this context, fenretinide-induced modulation of p-p38/pERK balance, together with repression of the mTOR pathway, is consistent with the induction of cellular dormancy. Cancer cell dormancy is increasingly recognized as a clinically relevant state that enables long-term persistence of DTCs, thereby contributing to minimal residual disease and tumor relapse.

### Modulation of the TME by fenretinide

**Figure 3:**
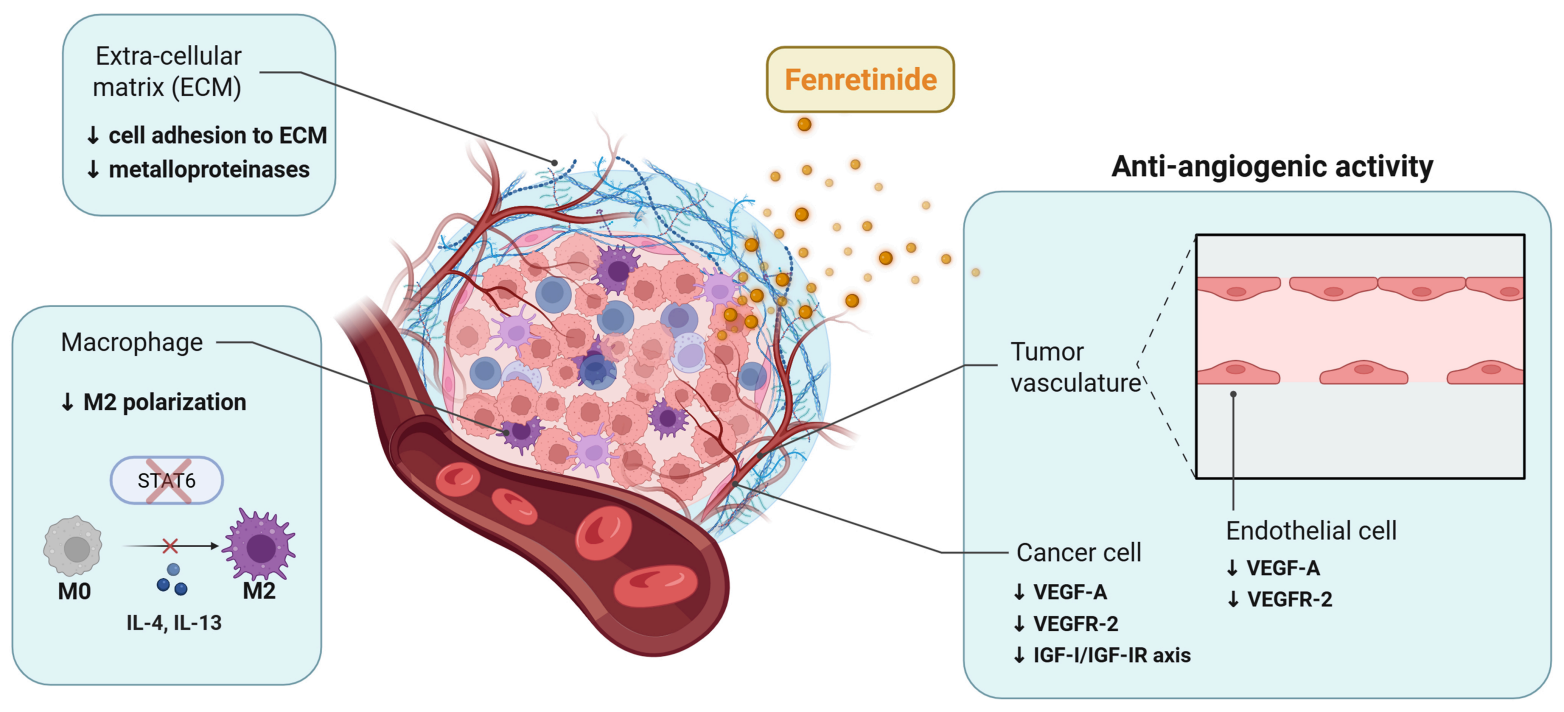
Modulation of the TME by fenretinide. Fenretinide shapes the TME by inhibiting angiogenesis, reducing the content of pro-tumor M2 macrophages, and disrupting tumor cell-ECM interactions. Blue boxes indicate the main effects of fenretinide on the non-cellular and cellular components of the TME. Created in BioRender. Verachi, P. (2026) https://BioRender.com/ugvfbl3. TME: Tumor microenvironment; ECM: extracellular matrix; STAT: signal transducer and activator of transcription; IL: interleukin; VEGF-A: vascular endothelial growth factor-A; VEGFR-2: VEGF receptor-2; IGF-I: insulin-like growth factor I; IGF-IR: IGF-I receptor.

A growing body of evidence shows that fenretinide is capable of modulating the TME through perturbation of signaling kinases and cell-extracellular matrix (ECM) interactions, inhibition of angiogenesis, and regulation of immune cells [Figure 3]. In a recent study on oral squamous cell carcinoma (OSCC), treatment with fenretinide induced several cancer-preventive effects including inhibition of basement membrane invasion, suppression of anchorage-independent growth, disruption of actin cytoskeletal components and inhibition of the focal adhesion kinase, a crucial protein that confers invasive properties^[20]^. Using molecular docking and kinase assays, it was demonstrated that fenretinide binds with high affinity to the ATP-binding pocket of all three c-Jun N-terminal kinase (JNK) isoforms, thereby inhibiting of the JNK signaling pathway associated with epithelial–myoepithelial transition. Functionally, fenretinide treatment reduced OSCC cell adhesion to the ECM and activation of matrix metalloproteinases (MMPs) [Figure 3]. In an *in vivo* OSCC model, local treatment with biodegradable polymeric implants containing fenretinide significantly downregulated the expression of the proto-oncogene *ERG*, an epithelial marker strongly associated with angiogenesis^[20]^. Consistent with these findings, other studies have demonstrated that fenretinide exerts robust anti-angiogenic effects through both direct and indirect mechanisms, reducing neovascularization and thereby enhancing antitumor efficacy^[19]^. In multiple myeloma (MM), treatment with fenretinide inhibited cell growth of cancer cells in their bone marrow microenvironment, both directly through induction of apoptosis and indirectly through inhibition of osteoclastogenesis and angiogenesis. Consistently, fenretinide inhibited tube formation by human umbilical vein endothelial cells (HUVEC) stimulated by either a cocktail of pro-angiogenic growth factors or by conditioned media derived from MM cells^[102]^. In a rat model of prostate cancer, fenretinide treatment significantly decreased carcinogenesis and reduced tumor vascularization^[103]^. Similarly, in a xenograft model using Y79 RB cells, fenretinide inhibited tumor growth through a robust reduction of microvessel formation. These findings were confirmed in *in vivo* Matrigel sponge assays, where fenretinide inhibited neovascularization induced by pro-angiogenic factors^[104]^. In Kaposi’s sarcoma, a highly vascularized tumor type, oral administration of fenretinide inhibited ectopic tumor growth and significantly reduced vascular density^[105]^. At the molecular level, fenretinide exerted its anti-angiogenic effects through down-regulation of the vascular endothelial growth factor (VEGF) signaling axis, a crucial pathway that drives angiogenesis. Specifically, the compound was able to reduce the expression levels of both VEGF-A and its receptor VEGFR-2 in endothelial and Kaposi’s sarcoma cells^[105]^ [Figure 3]. Furthermore, in a mouse model of hepatocarcinogenesis VEGF-A was found downregulated in pre-neoplastic liver lesions following fenretinide treatment, thereby impairing progression of the lesions to cancer^[106]^.

Several studies have linked the anti-angiogenic properties of fenretinide to its interaction with RARs, particularly RARα and RARβ^[19]^. Other studies have suggested that fenretinide is able to target the insulin-like growth factor-I (IGF-I)/IGF-I receptor (IGF-IR) axis, a key pathway promoting VEGF expression via hypoxia-inducible factor (HIF)-1α signaling. Consistent with this mechanism, treatment with fenretinide decreased plasma IGF-I levels in patients with breast and bladder cancer, downregulated IGF-I and IGF-IR in BC cells *in vitro* and suppressed IGF-I–mediated proliferation in meningioma and RB models^[19]^ [Figure 3]. Moreover, it has been demonstrated that fenretinide directly targets endothelial cells, interfering with multiple stages of neo-vessel formation. A mechanistic study showed that fenretinide reduces endothelial cell motility and invasiveness by inhibiting the activity of MMP-2, an enzyme essential for ECM degradation during vessel sprouting^[22]^. In addition, fenretinide upregulates bone morphogenetic protein-2 (BMP-2) and macrophage inhibitory cytokine-1 (MIC-1), two multifunctional cytokines with anti-angiogenic activity *in vitro* and *in vivo*. Notably, treatment with antibodies against BMP-2 counteracted the inhibitory effects of fenretinide, suggesting that suppression of angiogenesis is a key contributor to its antitumor activity^[22]^. Collectively, these findings indicate that fenretinide exerts a multifaceted inhibition of angiogenesis by acting on both tumor cells and components of the vascular TME while sparing the normal vasculature, as observed in clinical trials^[107]^. Interestingly, fenretinide has been shown to suppress IL-4/IL-13-mediated M2 polarization of RAW264.7 macrophages. This effect was associated with inhibition of phosphorylation of signal transducer and activator of transcription 6 (STAT6), a key regulator of M2 polarization [Figure 3]. Moreover, fenretinide inhibited tube formation by HUVEC cells exposed to conditioned media derived from M2 macrophages^[21]^. Consistent with these findings, in adenomatous polyposis coli (APC) transgenic mice, fenretinide inhibited colorectal tumorigenesis through a reduction of the M2-like macrophage population and a subsequent block of tumor angiogenesis^[21]^. Figure 3 and Table 1 summarize the effects of fenretinide on the TME, including its anti-angiogenic properties, inhibition of macrophage M2 polarization and suppression of cell-ECM interactions. Collectively, these data indicate that fenretinide actions create an unfavorable microenvironment for tumor progression, further supporting its potential as a chemopreventive and antitumor agent.

## CLINICAL EVALUATION OF FENRETINIDE IN CANCER

The antineoplastic and chemopreventive activity of fenretinide has been validated in several animal models, supporting its potential clinical application in cancer^[8,12,14,42,108]^. Clinically, fenretinide has been studied in phase I-III chemoprevention and chemotherapeutic trials primarily using an oral gelatin capsule formulation containing fenretinide suspended in corn oil and polysorbate 80 (National Cancer Institute fenretinide, NCI-FeR) [Table 2]. However, the results of these studies have shown high interpatient variability, primarily attributable to therapeutically effective drug concentrations not being consistently reached within tumor tissues. Pharmacokinetic studies reported that, even with dose escalation, plasma concentrations and tumor response rates did not increase proportionally, as would have been expected^[39,40,109]^. The variable and insufficient absorption of fenretinide can be attributed to its unfavorable pharmacokinetic profile: the low aqueous solubility and high lipophilicity of the molecule hinder its absorption and systemic distribution, ultimately restricting its penetration into tumor tissues^[44]^. Cooper *et al.* estimated a mean oral bioavailability of 16% in beagle dogs given a single 10 mg/kg oral dose of capsules, equivalent to a human dose of approximately 200 mg/m^2^^[42,110]^. In recent years, new fenretinide formulations have been developed in order to increase plasma concentrations of the drug, thereby potentially improving clinical responses (Table 3 and section “Novel fenretinide formulations: preclinical studies and clinical evidence”).

**Table 2:** Summary of clinical studies evaluating the effects of standard fenretinide formulations in cancer

**Formulation**	**Delivery method**	**Key advantages**	**Main limitations**	**Tumor**	**Daily dose**	**Plasma and/or intratumor concentrations**	**Clinical trial phase**	**Clinical** **response**	**Ref.**
Soft gelatin capsule (NCI fenretinide)	Oral corn-oil and polysorbate 80 capsule	Low toxicity	Poor and highly variable bioavailability; limited efficacy; poor patient compliance	Neuroblastoma (*n* = 53)	100-4,000 mg/m^2^	Plasma: 1.3-12.9 µM	I	No objective response; 77% SD	[109]
				Neuroblastoma (*n* = 39), Ewing sarcoma (*n* = 5) and other (*n* = 10)	350-3,300 mg/m^2^ (MTD = 2,475 mg/m^2^)	Plasma: 9.9 µM at the MTD	I	1 CR, 43% SD (neuroblastoma); 1 SD (Ewing sarcoma); 1 SD (melanoma)	[39]
				Advanced renal cell carcinoma (*n* = 19)	1,800 mg/m^2^	Intratumor: 3.6-7.9 µM	II	No objective response; 37% with SD	[111]
				Small-cell lung cancer (*n* = 19)	1,800 mg/m^2^	Plasma: 7.4 +/- 4.25 µM	II	No objective response; 24% with SD	[112]
				Prostate cancer (*n* = 23)	1,800 mg/m^2^	NA	II	No objective response; 30% with SD	[113]
				Prostate cancer (*n* = 27)	1,800 mg/m^2^	NA	II	No objective response	[114]
				Ovarian cancer (*n* = 22)	400-800 mg	Plasma 1.4 µM at the highest dose	I-II	No objective response	[116]
				Ovarian cancer (*n* = 31)	1,800 mg/m^2^	Plasma: 3.1-12.5 μM	II	42% SD at ≥ 9 μM; OS at 18 months 66% at ≥ 9 μM	[117]
				Brain tumor (*n* = 22 AG; *n* = 23 GBM)	1,200 or 1,800 mg/m^2^	Plasma: 1,213 +/- 261 ng/mL at the 900 mg/m^2^ bid dosage	II	1 partial radiologic response; 29% SD (AG arm), 9% SD (GBM arm) at 6 months	[115]
				BC and melanoma (*n* = 31)	300-400 mg	NA	II	Limited activity: 6.7% mixed response; 26.7% SD	[118]
				BC (*n* = 872 fenretinide arm *vs. N* = 867 observation arm)	200 mg (5 years)	NA	III	50% reduction in second BC risk in premenopausal women (≤ 40 years)	[38]

SD: Stable disease; MTD: maximum tolerated dose; CR: complete response; NA: not available; OS: overall survival; AG: anaplastic gliomas; GBM: glioblastoma; BC: breast cancer.

**Table 3:** Summary of preclinical and clinical studies evaluating the effects of novel fenretinide formulations in cancer

**Formulation**	**Delivery method**	**Key advantages**	**Main limitations**	**Tumor**	**Daily dose**	**Plasma and/or intratumor concentrations**	**Clinical trial phase**	**Clinical** **response**	**Ref.**
Fenretinide/LSX	LYM-X-SORB™ lipid matrix	Increased plasma levels *vs.* oral capsules	Limited long-term compliance due to GI side-effects; poor patient compliance	Adults with solid tumors and lymphomas (*n* = 20)	800-1,300 mg/m^2^	Plasma: ≥ 10 µM on day 7 at 1,000 mg/m^2^/day; > 20 μM in 1 patient who received 24 cycles	I	1 SD (cutaneous T-cell lymphoma)	[120]
				Neuroblastoma (*n* = 29)	352-2,210 mg/m^2^	Plasma: 21 µM on day 6 at a dosing regimen of 1,700 mg/m^2^/day	I	29% SD, 13% CR	[121]
Fenretinide/LSX + ketoconazole		Increased plasma levels *vs.* Fenretinide/LSX alone		Neuroblastoma (*n* = 34)	Cohort 1: 1,500 mg/m^2^ Cohort 2: Fenretinide/LSX + 6 mg/kg ketoconazole	Plasma: 18.4 µM Cohort 2 *vs.* 11.7 µM Cohort 1	I	2 CR, 1 PR, 1 mixed response (MIBG CR), 14 SD, and 12 progressive disease	[122]
Intravenous fenretinide	Lipid emulsion (mixture of egg phospholipids, glycerin, alcohol, and soybean oil)	Circumvents GI absorption issues; enables controlled dosing	Reversible hypertriglyceridemia	Malignant solid tumors (*n* = 18)	905-1,414 mg/m^2^	Plasma: 7-15 µg/mL at 1,131 mg/m^2^		No objective response, 28% SD	[124]
				Peripheral T-cell lymphoma (*n* = 11)	905-1,810 mg/m^2^	Plasma: 50 μM at the MTD 1,280 mg/m^2^/day	I	Response rate of 82%: 2 CR, 2 unconfirmed PR, 5 SD at doses ≥ 905 mg/m^2^/day	[125]
ST-001 nanofenretinide	Intravenous infusion	Phospholipid suspension	Relapsed/refractory T-cell non-Hodgkin lymphoma	(*n* = 46) *estimated	1.25-640 mg/m^2^	NA	Ia/Ib (recruiting)	Ongoing study; clinical data not available yet	ClinicalTrials.gov ID: NCT04234048
			Relapsed/refractory small cell lung cancer	(*n* = 44) *estimated		NA	Ia/Ib (recruiting)	Ongoing study; clinical data not available yet	ClinicalTrials.gov ID: NCT06922539
NanoFEN	Nanomicelles (complexation with 2-hydroxypropyl β-cyclodextrin)	Increased plasma levels *vs*. oral capsules	Preclinical only; iv administration	Preclinical (lung and CRC xenografts)	10 mg/kg	Plasma Cmax: 730.06 (oral) and 6,932.6 ng/mL (iv) at 5 mg/kg (single administration)	NA	NA (preclinical studies only)	[8]
NanoFEN + lenalidomide	Nanomicelles (phospholipids and triglyceride mixed with hydroxypropyl β-cyclodextrin)	Increased drug delivery; superior antitumor response compared to the single agent	Preclinical only; iv administration	Preclinical (human neuroblastoma xenografts)	30 mg/kg	Plasma: 10.06 µM; intratumor: 54.47 µM	NA	NA (preclinical studies only)	[128,129]
Bio-nFeR	Ion-pair stabilized lipid matrix	Oral administration; increased plasma levels *vs.* oral capsules	Preclinical only	Preclinical (lung cancer, melanoma and colon cancer CSCs-derived xenografts; neuT mice)	100 mg/kg	Plasma: 9.2 µM *vs.* 1.0 µM for capsules; intratumor: 9.6 µM *vs.* 1.5 µM for capsules	NA	NA (preclinical studies only)	[12,14]

GI: Gastrointestinal; SD: stable disease; CR: complete response; PR: partial response; MIBG: metaiodobenzylguanidine; MTD: maximum tolerated dose; NA: not available; iv: intravenous; CRC: colorectal cancer; Bio-nFeR: bioavailable fenretinide nanoformulation; CSCs: cancer stem cells.

### Clinical studies with oral fenretinide capsules

#### Neuroblastoma

In a phase I trial, oral fenretinide capsules were administered to children with neuroblastoma once daily in a range of doses from 100 to 4,000 mg/m^2^ for 28 days followed by a seven-day drug holiday. Although no severe toxicity was observed at the highest dose, excessive amounts of corn oil caused intestinal discomfort and precluded additional dose escalation. Day 28 plasma levels ranged from 1.3 to 12.9 µM in a dose-dependent manner. While no partial or complete responses (CR) were observed, 77% of patients achieved stable disease (SD) with a median of 23 months, and some of them showed partial regression^[109]^. The Children’s Oncology Group reported the results from a phase I clinical trial of oral fenretinide in 54 children with high-risk solid tumors, including neuroblastoma (*n* = 39), Ewing sarcoma (*n* = 5)^[39]^. Doses ranging from 350 to 3,300 mg/m^2^ were tested for days 1 to 7 every three weeks. The maximum tolerated dose (MTD) of 2,475 mg/m^2^ yielded a plasma concentration of 9.9 µM. One CR and 13 patients with SD were observed among 30 assessable neuroblastoma patients. Similar outcomes were observed in one of five Ewing sarcoma patients and one melanoma patient^[39]^.

#### Renal cell carcinoma

In a phase II trial, patients with unresectable or metastatic renal cell carcinoma received 900 mg/m^2^ twice daily (1,800 mg/m^2^ per day) for seven days in a three-week cycle for a total of 76 cycles. While no objective responses were observed, 37% of patients achieved SD with a median duration of 5.8 months. Consistent with the limited activity observed in patients, intratumor levels of fenretinide measured in three patients (3.6-7.9 µM) were at the lower end of the therapeutic window^[111]^.

#### Small-cell lung cancer

In 19 patients with small-cell lung cancer receiving a dose of 900 mg/m^2^ twice daily, no objective responses were observed. However, four of 17 response-evaluable patients (24%) achieved SD after 2-17 cycles. Among 14 patients, the mean plasma concentration of fenretinide on day seven of cycle one was 7.4 µM^[112]^.

#### Prostate cancer

In a phase II trial of oral fenretinide in patients with biochemically recurrent prostate cancer, the compound was administered at a dose of 900 mg/m^2^ twice daily for one week every three weeks for one year. The primary endpoint was a prostate-specific antigen (PSA) response, defined as a reduction of ≥ 50% and ≥ 5 ng/mL from the pretreatment value. Although well-tolerated, oral fenretinide did not meet prespecified PSA response criteria. However, 30% of patients achieved SD, suggesting modest single-agent clinical activity^[113]^. A separate phase II study in hormone refractory prostate cancer patients confirmed the limited antitumor activity of fenretinide, with seven of 22 evaluable patients achieving biochemical SD (plasma PSA stabilization) after 17.7 months^[114]^.

#### Brain cancer

In a phase II study conducted in adults with recurrent malignant gliomas [22 patients with anaplastic gliomas (AG) and 23 patients with glioblastoma (GBM)], oral fenretinide was administered on days one to seven and 22 to 28 of six-week cycles at doses of 600 or 900 mg/m^2^ twice daily (1,200 and 1,800 mg/m^2^ per day, respectively). The trial was discontinued after the first stage due to the limited activity at the tested doses. However, 29% of patients in the AG arm and 9% of patients in the GBM arm achieved SD at six months, and one patient with AG receiving 1,800 mg/m^2^ achieved a partial radiologic response^[115]^.

#### Ovarian cancer

An early phase I-II trial of oral fenretinide capsules (400-800 mg/day) in patients with ascitic ovarian cancer administered for up to four weeks prior to surgery found no objective responses. Mean plasma concentrations reached a mean of 1.4 µM at the highest dose tested, while drug levels in malignant ascitic cells and tumor tissue were 50 and five times lower, respectively, than those observed in carcinoma cells exposed to 1.4 µM fenretinide^[116]^. In a subsequent phase II trial for recurrent ovarian cancer, 1,800 mg/m^2^ of fenretinide (divided into twice-daily dosing) did not produce objective responses; however, 42% of patients achieved SD for a median of 7.9 months. Plasma fenretinide concentrations ranged from 3.1 to 12.5 μM (*n* = 24). Clinical outcomes were positively associated with fenretinide plasma levels: progression-free survival (PFS) at six months was 42% in patients achieving plasma concentrations ≥ 9 μM, compared to 17% in those below this threshold, while overall survival (OS) at 18 months was 66% *vs.* 13%, respectively^[117]^.

#### BC

An early phase II study on 31 patients with advanced BC or melanoma treated with daily fenretinide capsules (300-400 mg/day) reported no partial or CRs^[118]^. In 1987, a large multicenter phase III trial conducted a long-term analysis of the efficacy of a 5-year treatment with fenretinide (200 mg per day) in reducing contralateral or second ipsilateral BC in 2,867 patients aged 30-70 years with early BC who received no systemic therapy after primary treatment. After 8 years, no difference was observed in contralateral or ipsilateral BC; however, a post-hoc analysis suggested a significant correlation between treatment and patient menopausal status (or age), showing a 35% reduction in second BC risk among premenopausal women (or those aged < 50 years) and an opposite trend in postmenopausal women (or those aged > 50 years)^[38]^. A median 14.6 years follow-up from 1,739 women aged 30-70 (872 in the fenretinide arm and 867 in the observation arm) demonstrated a marked chemopreventive effect in the premenopausal subgroup: among women aged ≤ 40 years the risk in second BC decreased by around 50% and the protective benefit persisted for more than a decade after treatment cessation^[38]^. In summary, although oral fenretinide capsules exhibited low toxicological profile and favorable tolerability, the poor solubility of the compound severely limited its systemic distribution. In most clinical studies plasma and/or intratumor concentrations of fenretinide remained below 10 µM, whereas preclinical data suggest that steady-state concentrations exceeding 10 µM are required to ensure biological activity and therapeutic efficacy^[42]^. However, dose escalation in pharmacokinetic studies was constrained by capsule burden and by poor patient compliance, prompting the development of improved formulations.

### Novel fenretinide formulations: preclinical studies and clinical evidence

#### Oral fenretinide/LSX

To improve the oral delivery of fenretinide, the fenretinide was incorporated into a LYM-X-SORB lipid matrix, a mixture of lysophosphatidylcholine, monoglyceride and free fatty acids specifically designed to improve the absorption of lipophilic drugs through the proximal intestine into the lymphatic system^[119]^. *In vivo* studies have demonstrated that fenretinide/LYM-X-SORB (Fen/LXS) oral powder reached significantly higher concentrations in both plasma and tumor tissue of BALB/c mice as compared with NCI-FeR^[119]^. Furthermore, Fen/LXS oral powder extended survival in two of three mice bearing human neuroblastoma xenografts, further supporting its clinical utility^[119]^. In a phase I dose-escalation study, 20 adults with refractory malignancies received Fen/LXS oral powder administered in two divided doses for seven consecutive days every 21 days. The MTD regimens were 1,000 mg/m^2^/day (when given as two daily doses) and 800 mg/m^2^/day (when given as three daily doses) for seven days every 21 days. However, seven of 20 patients discontinued treatment due to gastrointestinal side effects or poor palatability. Plasma drug levels exhibited interpatient variability across all the doses tested, with some patients reaching concentrations higher than 9 μM^[120]^. In a separate phase I study, Fen/LXS was administered to 32 patients with relapsed/refractory neuroblastoma at doses ranging from 352 to 2,210 mg/m^2^/day. At a dose of 1,700 mg/m^2^/day mean plasma concentrations reached 21 µM on day six. Among the 29 response-evaluable patients, six achieved SD (29%), while four with bone disease had CR (13%). Overall, the Fen/LXS oral powder achieved two- to six-fold higher plasma concentrations compared to the NCI standard formulation, with minimal toxicity and evidence of antitumor activity^[121]^. Subsequently, Fen/LXS was tested in combination with ketoconazole, an inhibitor of fenretinide metabolism, in two cohorts of patients with high-risk recurrent or resistant neuroblastoma^[122]^. Cohorts one and two received fenretinide/LXS at 1,500 mg/m^2^/day on days one to seven every three weeks, while patients in the second cohort additionally received oral ketoconazole (6 mg/kg/day) on the same schedule. Pharmacokinetic analysis showed that co-administration of ketoconazole significantly increased plasma drug levels (mean peak concentration on day 7: 18.4 µM *vs.* 11.7 µM). Among 16 evaluable patients in cohort one and 18 in cohort two, treatment responses included two CR, one partial response, one mixed response, 14 SD, and 12 progressive diseases; an additional four patients did not complete the first course. Across the 34 patients with evaluable disease, the median PFS was 4.1 months^[122]^. These findings were further supported by animal studies^[123]^.

#### Intravenous lipid emulsion of fenretinide

To overcome the poor patient compliance associated with oral fenretinide, an intravenous (iv) formulation was developed using a lipid emulsion composed of a mixture of egg phospholipids, glycerin, alcohol, and soybean oil. This formulation achieved significantly higher mean plasma concentrations of fenretinide in animal studies^[110]^. In a phase I study involving patients with malignant solid tumors, the iv formulation was administered as a continuous infusion for five consecutive days in 21-day cycles^[124]^. The formulation exhibited a manageable safety profile and reached significantly higher plasma steady-state concentrations of the active metabolite compared to capsule formulations. Although no objective responses were observed, five patients achieved SD lasting for 11 to 22 weeks^[124]^. In hematological malignancies, iv fenretinide (80-1,810 mg/m^2^/day) showed promising results with a response rate of 82% in patients with T-cell lymphoma. Specifically, among 11 response-evaluable peripheral T-cell lymphomas, two had CRs, two had unconfirmed partial responses, and five achieved SD at doses ≥ 905 mg/m^2^/day^[125]^.

#### Fenretinide nanoformulations: the road to the future?

Recently, novel nanoformulations based on phospholipid–liquid triglyceride mixtures have been developed to improve fenretinide delivery and bioavailability. Nanoformulations offer several advantages over traditional drug delivery methods. In tumors characterized by abnormal vasculature, intravenously administered nanoparticles circulate systemically and retain the drug payload until extravasation occurs through discontinuities of the endothelium of tumor capillaries, a process known as enhanced permeability and retention effect, leading to selective drug accumulation and release within tumor tissue. When administered orally, nanoparticles enhance solubilization and improve absorption of lipophilic drugs in the gastrointestinal tract, thus increasing systemic bioavailability and therapeutic efficacy^[8]^. In 2019, Orienti *et al.* developed a new fenretinide nanoformulation (NanoFEN) through salification and complexation with 2-hydroxypropyl β-cyclodextrin, a solubilizing excipient characterized by favorable biodistribution and minimal toxicity^[8]^. In mice, a single administration of NanoFEN (5 mg/kg) by oral or iv routes resulted in significantly higher plasma concentrations of fenretinide (Cmax: 730.06 and 6,932.6 ng/mL, respectively) as compared to those achieved with an equivalent dose of oral fenretinide capsules (Cmax: 298.13 ng/mL)^[8]^. In the same study, NanoFEN exerted a potent antitumor activity in cell lines derived from solid and hematologic tumors, in primary spheroid cultures and in xenograft models. Importantly, treatment with NanoFEN significantly inhibited the growth of lung and CRC xenografts, in the latter independently of the mutational status of the tumor cells. Moreover, a prolonged 70-day treatment in mice bearing lung cancer xenografts resulted in complete arrest of tumor growth, with no detectable signs of toxicity^[8]^, supporting the potential use of fenretinide as a dormancy-inducing strategy. Subsequently, the same group generated Bio-nFeR, a nanomicellar fenretinide formulation with the key advantage of enabling oral administration as a liquid suspension^[14]^. An extensive pharmacokinetic study revealed plasma concentrations of fenretinide 1.3 and 1.6 times higher with Bio-nFeR than with NCI-FeR oral capsules after initial and repeated dosing, respectively^[14]^. Moreover, chronic administration of Bio-nFeR resulted in a 40% increase in plasma concentrations as compared to day one^[14]^. In the same study, the antitumor activity of Bio-nFeR was assessed in patient-derived three-dimensional cultures and in xenograft models, in comparison with NCI-FeR. Bio-nFeR induced cytotoxicity in cancer cells even at low concentrations, while NCI-FeR produced only a moderate reduction in cell viability at the highest dosage tested. Consistently, Bio-nFeR significantly suppressed the growth of lung cancer xenografts, whereas NCI-FeR did not affect tumor growth. Finally, no signs of systemic toxicity were observed following repeated Bio-nFeR administration, supporting the feasibility of dose escalation in further preclinical studies^[14]^. In xenograft models derived from lung, CRC and melanoma CSCs, Bio-nFeR caused a significant arrest of tumor growth without evidence of systemic toxicity. In conclusion, Bio-nFeR achieved plasma concentrations above the therapeutic threshold and higher than those reported in other studies. This enhanced bioavailability was accompanied by superior antitumor efficacy *in vivo*, providing a strong rationale for the clinical translation of Bio-nFeR^[14]^. In a recent study using neuT mice, treatment with Bio-nFeR significantly delayed tumor onset and, most importantly, showed high efficacy against metastatic progression, inhibiting both metastasis initiation and expansion. These findings support the potential of Bio-nFeR for both prevention and treatment of metastatic BC^[12]^. Notably, the doses of Bio-nFeR used in animal models were comparable to the lower doses used in drug escalation clinical trials using other fenretinide formulations^[12,14]^. Specifically, 100 mg/kg in mice corresponds to 300 mg/m^2^ in humans, whereas clinical trials using other fenretinide formulations have safely tested doses up to 4,000 mg/m^2^ with manageable toxicity. These data suggest that Bio-nFeR dosing can be further escalated without significant adverse effects, potentially increasing its therapeutic efficacy. Future clinical studies are needed to validate the promising effects of Bio-nFeR as both a tumor-preventive and dormancy-inducing therapeutic agent. Fenretinide nanoformulations have also demonstrated potent antitumor activity and improved bioavailability in preclinical models of neuroblastoma and APL, further supporting their translational potential^[92,126-129]^. Finally, a phospholipid-based fenretinide nanoformulation for iv administration (ST-001) is currently being evaluated in phase I clinical trials for relapsed/refractory T-cell non-Hodgkin’s lymphoma (NCT04234048, active) and for relapsed/refractory non-small cell lung cancer (NCT06922539, recruiting). These trials aim to define parameters including MTD, dose limiting toxicity (DLT), objective response rate (ORR) and PFS. These studies may address the limitations of previous formulations by generating clinical evidence of improved pharmacokinetic profiles and patient outcomes associated with nanoformulations.

### Potential mechanisms of fenretinide resistance

There is no clear evidence to date that cancer cells develop resistance to fenretinide. While resistance to ATRA is well documented and involves diverse mechanisms, such as RARα mutations and activation of compensatory survival pathways, fenretinide has shown efficacy even in ATRA-resistant tumor cells^[130]^. Nevertheless, potential adaptive or intrinsic mechanisms of resistance to fenretinide should be considered and evaluated in future studies. Such mechanisms may involve:

(a) **Alterations in the apoptotic protein network**, such as upregulation of anti-apoptotic Bcl-2 family proteins or suppression of Bcl-2 homology 3 domain (BH3)-only proteins. In this regard, preclinical studies on the synergistic activity of fenretinide in combination with BH3 mimetics suggest diverse and complementary actions of the two drug types^[131,132]^.

(b) **Enhanced expression of ROS scavengers**. Interestingly, a study on tumors of the Ewing sarcoma family demonstrated that the intracellular glutathione antioxidant system is a critical determinant of cellular sensitivity to fenretinide^[133]^.

(c) **Bypassing mTOR inhibition**. Since fenretinide inhibits mTOR activity, mechanisms of resistance to mTOR inhibitors, including activation of compensatory kinases, feedback activation of PI3K/AKT signaling or mTOR mutations affecting drug binding^[134]^, may theoretically block fenretinide effects on the mTOR pathway.

(d) **Drug efflux mechanisms**. Multidrug resistance efflux transporters and other mechanisms such as membrane rigidification can regulate intracellular fenretinide levels^[92]^. However, in Ewing sarcoma cells fenretinide is not a substrate of the ATP-binding cassette (ABC) transporter multidrug resistance-associated protein 1 (MRP1), underscoring tumor–specific differences in the contribution of drug efflux proteins to fenretinide sensitivity^[135]^.

In summary, while the multifaceted mechanism of action of fenretinide makes the development of resistance less likely, tumor cells may theoretically still acquire escape mechanisms. Future studies evaluating novel fenretinide formulations will be crucial to identify and overcome potential resistance mechanisms.

## CONCLUSIONS AND FUTURE PERSPECTIVES

**Figure 4:**
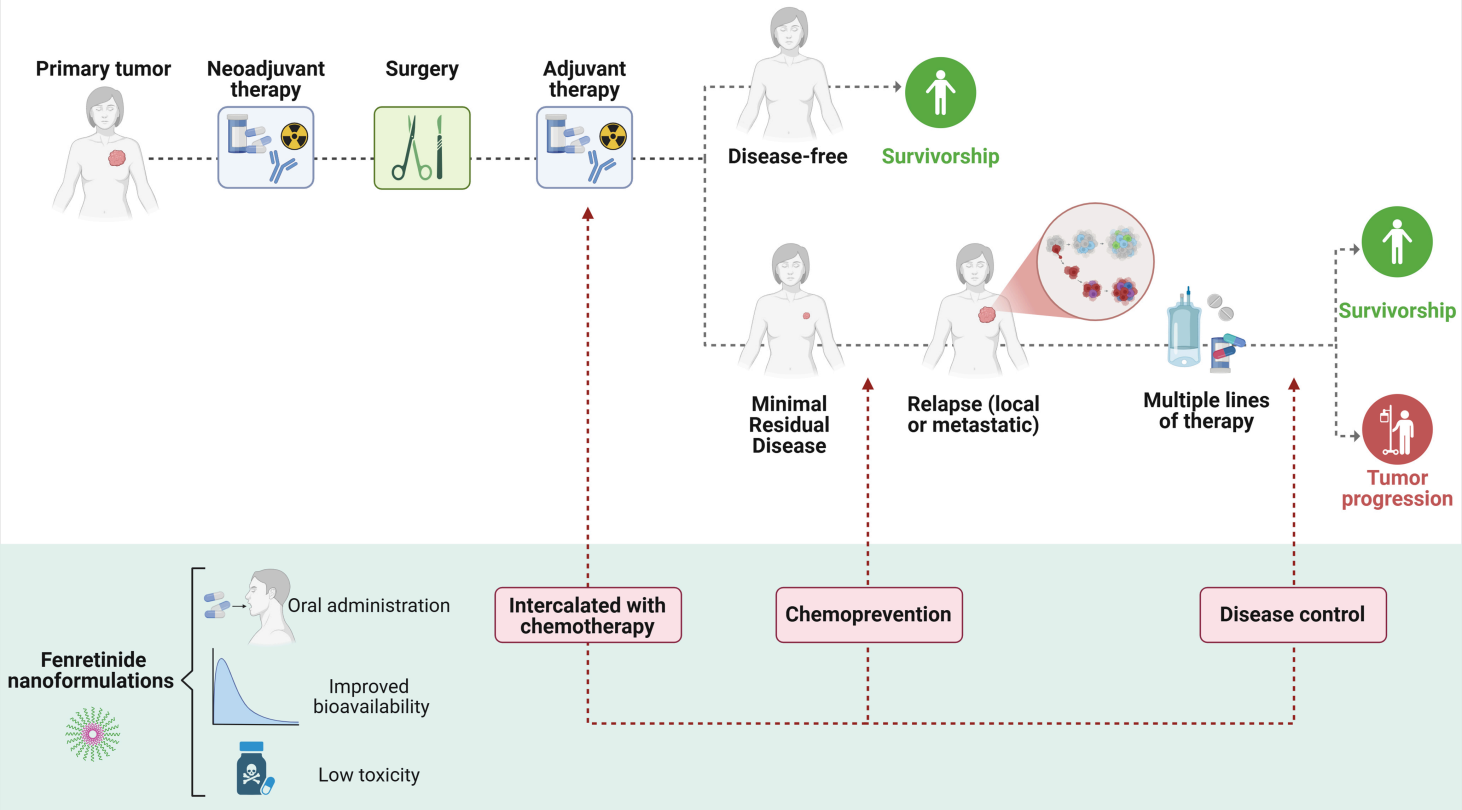
Clinical opportunities for fenretinide in cancer. Schematic overview of possible applications of novel fenretinide nanoformulations in the clinical management of cancer patients. Potential uses of the compound at various stages of patient treatment are suggested: as a chemopreventive agent, as part of standard therapeutic regimens intercalated with chemotherapy agents, or for long-term disease control in advanced tumors. Created in BioRender. Verachi, P. (2026) https://BioRender.com/mu883r2.

Fenretinide has long been investigated for its pleiotropic antitumor properties and favorable toxicity profile. Unlike classic retinoids, which primarily act through nuclear RARs, fenretinide exerts its activity through both RAR-dependent and RAR-independent mechanisms, resulting in a broad spectrum of biological effects, including induction of apoptosis, ROS production and inhibition of biosynthetic pathways. Notably, fenretinide has been consistently shown to display marked anti-proliferative activity and the ability to reduce the population of stem-like cells, which are increasingly recognized as major drivers of therapeutic resistance and disease recurrence. More recently, preclinical studies in animal models of metastatic BC have shown that fenretinide is able to induce cellular dormancy, thereby suppressing both the initiation and progression of metastases. Despite extensive preclinical evidence supporting its efficacy, early clinical trials with oral formulations demonstrated only limited antitumor activity in multiple solid and hematological tumors. This discrepancy between preclinical observations and clinical data is likely attributable to pharmacokinetic limitations, as multiple studies have demonstrated that plasma and/or intratumoral concentrations of fenretinide achieved with the standard formulation often failed to reach the threshold required for cytotoxic activity. As previously mentioned, fenretinide exhibits physicochemical properties distinct from those of other retinoids such as ATRA, including marked lipophilicity, which limits its bioavailability. This difference in pharmacokinetic behavior highlights the particularly critical challenge posed by fenretinide’s limited systemic exposure and underscores the need for advanced formulation strategies to improve its clinical efficacy. In recent years, novel formulations based on lipid-based oral suspensions and nanoencapsulated delivery systems have significantly improved fenretinide delivery and bioavailability, thus renewing interest in its clinical applications. By reducing dose requirements, nanoformulations may also mitigate gastrointestinal adverse effects and improve patient compliance, which were limiting factors in early clinical trials. Consistent with this, preclinical *in vivo* studies using fenretinide nanoformulations have reported absence of weight loss, inappetence or other signs of gastrointestinal distress, suggesting improved tolerability as compared with earlier oral formulations^[12,14]^. Moreover, early-phase clinical evaluation of ST-001 nanofenretinide has demonstrated an excellent tolerability profile with no unexpected toxicities reported to date. However, systematic assessment of gastrointestinal safety in patients is still lacking^[136]^. Future strategies, such as the addition of flavoring agents, could further enhance the palatability of oral fenretinide formulations and facilitate their clinical translation. The recent development of novel fenretinide formulations offers a promising opportunity to facilitate its clinical translation. The multifaceted pharmacological activity of fenretinide supports its potential integration at multiple stages of cancer management. Figure 4 illustrates possible applications of fenretinide nanoformulations to cancer treatment schedules, specifically (i) chemoprevention in tumors with high risk of relapse after surgery and/or chemotherapy/radiotherapy; (ii) disease stabilization in progressing tumors; and (iii) intercalation schedule between chemotherapy cycles. First, the use of fenretinide as a chemopreventive agent is consistently supported by data from various preclinical models demonstrating its ability to promote cell cycle arrest and maintain tumor cells in a non-proliferative cell cycle state. Dormant micro-metastases or single dormant DTCs are widely recognized as key contributors to late relapse in tumors with high risk of recurrence, such as BC^[137]^. Although the majority of BC patients are diagnosed at an early, potentially curable stage, 20%-30% eventually experience recurrence due to occult minimal residual disease^[137]^. Subsets of patients at high risk of late relapse can be identified through specific patterns of genomic copy-number alterations and gene expression^[138]^, making them eligible for therapies aimed at preventing disease recurrence. The importance of targeting dormant cancer cells to prevent BC recurrence is illustrated by a recent randomized phase II trial using hydroxychloroquine, the mTOR signaling inhibitor everolimus or their combination in BC survivors within 5 years of diagnosis who had detectable DTCs in bone marrow aspirate^[139]^. The ability of fenretinide to induce a dormancy phenotype highlights its potential as a chemopreventive strategy in cancer patients following surgical tumor resection with the aim of reducing the risk of disease recurrence. This concept is reinforced by a recent study showing that the novel formulation Bio-nFeR inhibited metastatic progression in neuT mice^[12]^. In addition, clinical trials using conventional oral formulations have shown a significant reduction in the risk of second BC in premenopausal women, with the protective effect persisting after treatment discontinuation^[38]^. These observations highlight a potential window of opportunity to evaluate modern nanoformulations in the adjuvant or post-adjuvant setting, where the primary objective is long-term suppression of minimal residual disease. This approach reflects the evolving paradigm of reducing recurrence risk by targeting specific biological features of dormant cancer cells. Moreover, the low-toxicity profile of fenretinide supports its suitability for chronic administration, a critical consideration in malignancies such as BC, where late relapse may occur 20-30 years after primary diagnosis^[140]^. Second, fenretinide may be considered for the management of advanced and rapidly progressing tumors with limited or no therapeutic options, where disease stabilization represents a clinically relevant endpoint. The historical observation that treatment with fenretinide often resulted in SD rather than in objective responses across multiple tumor types may actually represent a therapeutic goal in this context. Furthermore, the favorable safety profile of fenretinide makes this compound suitable for cancer patients debilitated by both tumor burden and prior intensive pharmacological or radiation therapies, rendering them more vulnerable to treatment-related toxicities. Third, we propose that novel fenretinide nanoformulations could be integrated into chemotherapy regimens, either in combination or in alternation to conventional cytotoxic agents. Substantial evidence indicates that standard chemotherapy, while restraining the expansion of responsive tumors, paradoxically enriches for CSCs that are responsible for chemoresistance and relapse^[24]^. In this context, the ability of fenretinide to target CSCs and downregulate stemness-related genes may be exploited in a clinical setting to prevent chemotherapy-induced progression. Based on this hypothesis, integration of fenretinide between chemotherapy cycles would ideally function as a “biological brake”, counteracting the rebound effects of chemotherapy and extending the durability of clinical benefit. Although not yet validated in the clinic, this hypothesis is supported by substantial preclinical evidence and warrants systematic evaluation in future studies. Indeed, several preclinical studies have already demonstrated that fenretinide can enhance the efficacy of conventional chemotherapeutic and immunotherapeutic agents. For example, Formelli *et al.* showed that fenretinide enhances cisplatin sensitivity in both cisplatin-sensitive and cisplatin-resistant ovarian tumor models^[141]^. Similarly, Kalemkerian *et al.* reported that fenretinide in combination with cisplatin, etoposide, or paclitaxel produced more-than-additive growth inhibition of a small cell lung cancer cell line, indicating a potential pharmacological synergy^[142]^. Other preclinical studies have shown that fenretinide in combination with bortezomib, selenite, ABT-263 (Navitoclax), histone deacetylase inhibitors or CD20-directed antibodies exhibited enhanced efficacy in multiple cancer types including melanoma, ovarian cancer, neuroblastoma and hematologic malignancies. Compared to single agents, drug combinations resulted in increased apoptosis, ROS accumulation, mitochondrial dysfunction and suppression of survival pathways^[143-150]^. Recently, Orienti *et al.* have shown complete regression of tumor growth in neuroblastoma xenograft models treated with fenretinide nanomicellar formulations combined with the immunomodulatory drug lenalidomide^[128,129]^. These findings suggest that fenretinide may synergize with immunomodulatory agents by remodeling the TME, thereby potentiating anti-angiogenic effects and increasing tumor cell susceptibility to immune-mediated responses. In this context, investigating a potential synergy with immune checkpoint inhibitors may be of interest to further increase tumor cell susceptibility to immune-mediated cytotoxicity. Despite promising preclinical evidence, clinical evaluations of traditional fenretinide formulations^[151-153]^ in combination therapies have shown acceptable tolerability but limited efficacy, reflecting suboptimal systemic exposure. The development of novel fenretinide formulations may offer a promising strategy for overcoming these limitations by achieving higher and more sustained drug levels, thereby potentially amplifying synergistic interactions with other chemotherapeutic agents. Translating the potential of combination therapies into clinical practice will require additional preclinical studies, careful design of intercalated schedules and dosing optimization for fenretinide nanoformulations. Furthermore, the identification of biomarkers potentially predictive of response to fenretinide, such as mTOR pathway hyperactivation, expression of CSCs markers or expression of factors specifically associated to lipid metabolism, could enable stratification of patients eligible for fenretinide treatment, thus maximizing clinical benefits. In conclusion, this review highlights the pleiotropic biological effects of fenretinide and underscores the potential clinical relevance of its novel formulations. The preclinical and clinical evidence discussed herein supports the view that fenretinide should not be considered a conventional cytotoxic drug but rather a disease-modifying agent capable of attenuating tumor aggressiveness by promoting dormancy and reducing stemness. This perspective aligns with the evolving paradigm in modern oncology that emphasizes chronic disease management and therapeutic strategies targeting minimal residual disease. Taken together, fenretinide’s unique pharmacological profile together with the development of novel formulations that improve bioavailability, encourage a clinical reassessment of this drug in both therapeutic and chemopreventive settings. Novel fenretinide formulations may potentially address unmet clinical needs such as relapse prevention in high-risk patients or the management of advanced or metastatic tumors, filling a gap where conventional therapeutic approaches provide limited results.

## DECLARATIONS

### Copyright

© The Author(s) 2026.
